# Rethinking ethical governance of generative AI in sport pedagogy research: a discipline-sensitive perspective

**DOI:** 10.3389/fpubh.2026.1812516

**Published:** 2026-05-08

**Authors:** Amayra Tannoubi, Vlad Adrian Geantă, Viorel Petru Ardelean, Edi Setiawan, Fairouz Azaiez

**Affiliations:** 1 High Institute of Sport and Physical Education of Gafsa, University of Gafsa, Gafsa, Tunisia; 2 Sports Performance Optimization Research Laboratory (LR09SEP01), National Center for Sports Medicine and Science (CNMSS), Tunis, Tunisia; 3 Department of Physical Education and Sport, Faculty of Physical Education and Sport, Aurel Vlaicu University of Arad, Arad, Romania,; 4 Research Center for Physical Activities, Arad, Romania; 5 Faculty of Teacher Training and Education, Suryakancana University, Cianjur, Indonesia

**Keywords:** AI disclosure, generative artificial intelligence, higher education, motor learning, pedagogical validity, research ethics, sport pedagogy

## Abstract

Generative artificial intelligence (GenAI)—including large language models, image synthesis, and learning analytics tools, is increasingly used in pedagogical research for instructional design, feedback generation, data analysis, and manuscript preparation. However, discipline-specific ethical guidance remains limited, particularly in fields characterized by embodied learning and performance-based pedagogy. In sport sciences, GenAI applications intersect with motor learning, sensorimotor feedback, and learner autonomy, raising distinctive challenges related to academic integrity, transparency, authorship, data protection, and pedagogical validity. In this Perspective, we argue that existing institutional and international AI ethics frameworks, while essential, remain predominantly text-oriented and insufficiently responsive to the embodied, safety-sensitive, and movement-based characteristics of sport pedagogy research. This creates a governance gap in which AI-generated feedback may directly affect motor learning processes, learner safety, and skill acquisition trajectories in ways not adequately captured by generic AI ethics guidance. Drawing on research integrity standards, global AI governance principles, and motor learning theory, we identify key ethical tensions emerging from the integration of generative systems into sport pedagogical research and practice. This article develops a discipline-sensitive ethical framework through a structured, purposive, and theory-guided synthesis of relevant literature. We propose a set of priority orientations to support responsible GenAI use in this context, including strengthened human accountability, transparent disclosure practices, discipline-informed validation of AI-generated feedback, and enhanced protection of learner data. These propositions require empirical validation in real world sport pedagogy contexts. We position pedagogical validity, defined as alignment between AI-supported processes and established motor learning principles, as a novel ethical criterion not yet explicitly articulated in AI education governance. Finally, we outline a research and policy agenda for empirically validating this framework across diverse embodied learning environments and clarify the logic used to identify and synthesize literature across AI governance, research integrity, and motor learning scholarship. This conceptual, theory-guided Perspective is intended to inform researchers, ethics committees, and higher education institutions seeking disciplines-sensitive approaches to the responsible integration of GenAI in embodied learning and performance-based education contexts.

## 1 Introduction

Generative artificial intelligence (GenAI) systems capable of producing text, analytical summaries, instructional materials, and feedback are rapidly reshaping research and pedagogical practices in higher education. Across disciplines, these systems are increasingly integrated into manuscript preparation, instructional design, formative assessment, and data organization (1). Recent research also documents growing adoption of AI-supported analytics and feedback systems within physical education and sport training environments (2). International bodies such as the OECD (3), European Commission (4), Holmes and Maio (5), and major publishers including Frontiers (6) have issued high-level guidance emphasizing transparency, accountability, and human oversight in AI use. However, existing ethical frameworks remain largely discipline-neutral and predominantly oriented toward text-based, cognitively centered domains of scholarship (7, 8). They rarely address the specific epistemological and pedagogical characteristics of embodied disciplines such as sport sciences where learning emerges through perception–action coupling, task–environment interaction, and adaptive motor exploration (9–11).

This governance gap is particularly acute in sport pedagogy because AI-generated feedback operates performatively: it does not merely convey information but directly shapes motor coordination, attentional allocation, and movement exploration patterns during skill acquisition (12, 13). For example, AI feedback promoting excessive internal attentional focus (e.g., “bend your knees more”) may conflict with evidence favoring external focus instructions (e.g., “push the ground away”), potentially degrading rather than supporting learning (14). Similarly, algorithmically generated feedback lacking contextual sensitivity to task constraints may inadvertently constrain adaptive movement variability essential for skill development within a constraints-led framework (9, 15). These pedagogical consequences extend beyond the academic integrity concerns that dominate current AI ethics discourse.

Sport pedagogy research also frequently involves sensitive data including video recordings, biomechanical measures, and performance analytics that raise heightened concerns regarding data protection and informed consent when processed through cloud-based GenAI systems (16). For instance, wearable sensors and video-based pose estimation systems used in physical education increasingly transmit identifiable biomechanical data, movement patterns, and performance metrics to cloud-based AI platforms for analysis and feedback generation (17, 18). Such data may reveal not only athletic performance, but also physical capabilities, movement dysfunctions, and health-related information subject to enhanced legal and ethical protection. Although general AI governance frameworks emphasize responsible data use, they do not sufficiently operationalize how these principles should be implemented in embodied learning environments where feedback precision, contextual sensitivity, and learner safety are central (19).

This limitation is evident in widely cited governance guidance. Holmes and Miao (5) emphasize transparency and human oversight in educational uses of generative AI, while the Organisation for Economic Co-operation and Development (3) stress trustworthiness and accountability. Yet such frameworks do not specify how ethical evaluation should proceed in contexts where AI feedback directly influences physical coordination, attentional focus during movement execution, or learner autonomy in motor exploration. At the same time, research on AI-assisted coaching and movement analysis shows that automated systems are increasingly used for real-time movement correction, biomechanical analysis, and performance evaluation (20–23). This disconnects between generic AI ethics frameworks, and the realities of embodied learning creates a governance gap that the present article seeks to address.

We therefore argue that the ethical challenge in sport pedagogy extends beyond academic integrity toward what we term pedagogical validity, a discipline-specific ethical criterion that, to our knowledge, has not been explicitly articulated in existing AI education governance frameworks. We introduce pedagogical validity as a discipline-specific ethical criterion (see Framework section for full operationalization) (9, 10, 12, 13). This criterion is necessary because AI systems that appear ethically acceptable from transparency or authorship perspectives may nevertheless undermine learning if they conflict with established mechanisms of skill acquisition.

This article develops a discipline-sensitive ethical framework through a structured, purposive, and theory-guided synthesis of literature across AI governance, research integrity, and motor learning. Because the aim is conceptual development rather than exhaustive evidence mapping, the review process should be understood as purposive and interpretive, not systematic in the PRISMA sense. To reduce selection ambiguity, sources were retained when they directly informed at least one of the following questions: what ethical principles govern AI use, what responsibilities frame scholarly integrity, and what pedagogical conditions support valid motor learning. By synthesizing ethical governance principles with motor learning theory, the paper seeks to stimulate disciplinary reflection and guide responsible practice rather than to evaluate specific technologies or interventions.

This Perspective makes three interrelated contributions to the emerging discourse on generative AI in education. First, it differentiates AI ethics in text-based academic domains and embodied disciplines such as sport pedagogy. Second, it introduces and operationalizes the concept of pedagogical validity as a necessary ethical condition for GenAI use in motor learning research contexts. Third, it proposes a discipline-specific ethical decision framework that integrates research integrity standards, AI ethics in education, and motor learning theory to guide responsible GenAI use in sport pedagogy research. The framework does not claim that all GenAI uses in sport pedagogy pose equal ethical risk; rather, risk is expected to vary according to pedagogical function, degree of automation, data sensitivity, and the possibility of human expert review. Rather than seeking to restrict technological innovation, this paper aims to articulate boundaries within which GenAI can be integrated responsibly, ensuring that technological augmentation remains aligned with learner-centered, evidence-based pedagogical principles and the irreplaceable expertise of human coaches and researchers.

By anchoring AI ethics within motor learning theory and constraints-led pedagogy, we propose a framework that moves beyond abstract principles toward discipline-sensitive guidance for research contexts subject to formal ethical review. In doing so, we seek to contribute to the broader conversation on responsible AI integration in education by foregrounding the ethical complexities of embodied learning environments.

## 2 Conceptual foundations for ethical GenAI use in sport pedagogy

Ethical evaluation of GenAI in sport pedagogy requires integration of research integrity standards, AI ethics in education, and motor learning theory. Research integrity frameworks emphasize authorship responsibility, transparency, and accountability (24, 25). These standards clarify that responsibility for intellectual content cannot be delegated to algorithmic systems. However, such guidance primarily addresses scholarly authorship and publication ethics, offering limited insight into pedagogical consequences in embodied learning contexts.

AI ethics in education extends the analysis by foregrounding human-centered design, transparency, and learner protection (3, 26). These frameworks stress that AI systems must augment rather than replace human expertise. Yet they typically conceptualize learning in cognitive or informational terms, with insufficient attention to domains where feedback directly shapes physical coordination, perception–action coupling, and learner autonomy.

In this context, algorithmically generated feedback that lacks contextual sensitivity may constrain exploration or promote maladaptive movement solutions (9, 10, 12, 13). Evidence on attentional focus shows that instructions directing attention toward body movements tend to produce inferior learning and performance relative to instructions emphasizing movement effects on the environment (14, 27). Similarly, excessive augmented feedback can create dependency and undermine learner autonomy, whereas effective learning requires room for exploration and adaptive adjustment (12). From a constraints-led perspective, AI-generated feedback that prescribes a single “correct” solution may inhibit the discovery of individualized, functional coordination patterns (9, 11).

Integrating these domains reveals that ethical AI use in sport pedagogy cannot be evaluated solely through transparency or authorship criteria. It must also be assessed in relation to established mechanisms of motor learning. We therefore argue that pedagogical validity should function as a core ethical requirement in embodied learning research. This reveals a broader governance gap: research integrity standards establish that humans remain accountable, but they do not specify what counts as appropriate validation for movement-based feedback; AI ethics in education emphasizes learner protection, but it often conceptualizes learners primarily as cognitive agents; and motor learning theory identifies mechanisms of skill acquisition, but it has not yet been systematically integrated into AI ethics discourse. Addressing this gap requires not merely adapting existing frameworks but fundamentally reconceptualizing AI ethics through the lens of embodied learning theory.

## 3 A discipline-specific ethical framework

Drawing from these foundations, we propose a discipline-specific ethical framework for evaluating GenAI use in sport pedagogy research. This framework is conceptual and intended to support ethical reflection and structured decision-making rather than function as a perspective regulatory model. For that reason, the framework is intended as an evaluative scaffold for structured ethical judgment, not as a substitute for empirical evidence, institutional policy, or expert pedagogical review.

### 3.1 Human responsibility

Human researchers and qualified coaches retain ultimate accountability for research design, instructional decisions, and interpretive judgments. While GenAI may assist with drafting or organizing materials, it cannot assume epistemic or ethical responsibility. In embodied learning contexts, final authority must remain with domain experts capable of evaluating contextual appropriateness. In practice, this means that any AI-generated instructional recommendation affecting task design, movement correction, or learner evaluation should be reviewed and explicitly endorsed by a qualified human expert before implementation.

### 3.2 Transparency

All GenAI use should be transparently disclosed in ethics applications and scholarly publications. Transparency enables informed consent, peer scrutiny, and institutional oversight. Beyond tool disclosure, researchers should clarify the scope of use, and the level of human review applied. At minimum, disclosure should specify the tool used, its functional role, the stage of the research process in which it was used, and the extent of human verification applied to its outputs.

### 3.3 Academic integrity

GenAI may support organizational or linguistic processes but should not substitute for theoretical reasoning, hypothesis formation, or scientific interpretation. Intellectual contributions must remain traceable to human authors. In sport pedagogy research, this includes pedagogical decision-making and feedback design.

### 3.4 Data protection

Sport pedagogy research frequently involves identifiable performance data, video recordings, and biometric information. Ethical AI integration requires compliance with applicable data protection legislation and explicit informed consent where AI systems process participant data. Special caution is warranted when using cloud-based platforms. Where video, biomechanical, or wearable-derived data are involved, researchers should additionally document data storage location, third-party processing exposure, retention period, and participant consent language specific to AI-supported analysis.

### 3.5 Pedagogical validity

Pedagogical validity represents this framework’s core conceptual contribution and distinguishes embodied learning contexts from predominantly cognitive domains. We define pedagogical validity as the alignment between AI-supported instructional processes and empirically grounded motor learning principles, operationalized through four interrelated dimensions:

1. Attentional Focus Appropriateness: AI-generated feedback should promote external rather than internal attentional focus, consistent with robust evidence that external focus enhances both learning and performance (14, 27). Feedback must be evaluated according to whether it directs learners’ attention to movement effects (external) or body movements themselves (internal).
2. Autonomy Support: AI systems should preserve rather than constrain learner autonomy and self-directed exploration. Excessive prescriptive feedback or rigid movement templates may undermine intrinsic motivation and adaptive learning processes (12, 13).
3. Context Sensitivity: Feedback must account for task constraints, environmental conditions, and individual performer characteristics rather than applying generic movement prescriptions. From a constraints-led perspective, effective movement solutions emerge from performer-task-environment interactions (9, 10).
4. Movement Variability Support: AI systems should support rather than suppress functional movement variability. Variability during practice facilitates exploration of perceptual-motor workspace and stabilization of coordination patterns, whereas feedback promoting a singular “optimal” forms may constrain this exploratory process (9, 15).

Pedagogical validity cannot be assumed or inferred from technical performance metrics such as classification accuracy, because technically accurate output is not necessarily pedagogically appropriate. To our knowledge, this criterion has not been explicitly articulated in existing AI education governance frameworks, despite its centrality to embodied learning contexts where feedback directly shapes skill acquisition trajectories.

### 3.6 Distinguishing discipline-sensitive from generic AI ethics governance

This framework extends beyond adaptation of generic AI ethics principles in three substantive ways. First, it integrates domain-specific learning theory as a necessary component of ethical evaluation, arguing that pedagogical validity constitutes an ethical requirement rather than merely a quality indicator. Second, it positions safety concerns not as abstract risks but as concrete consequences of feedback that violates established motor learning principles for example, feedback promoting maladaptive movement patterns that increase injury risk. Third, it operationalizes ‘human oversight’ not as generic review but as expert validation by motor learning specialists capable of evaluating feedback against constraints-led pedagogy and attentional focus research. These features distinguish the framework from generic AI governance approaches that, while emphasizing similar values (transparency, accountability, human oversight), lack the disciplinary specificity required for embodied learning contexts.

## 4 Decision pathway for responsible use

**Figure 1:**
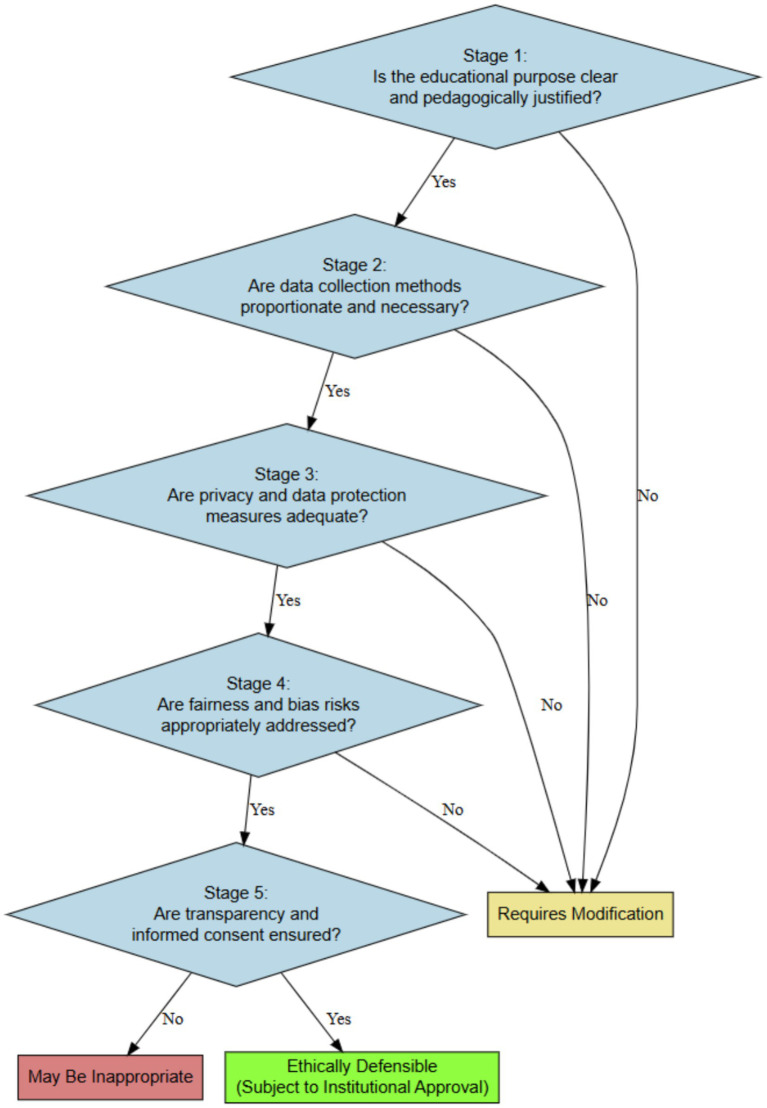
Ethical decision pathway for evaluating GenAI use in sport pedagogy research. The pathway guides researchers through sequential decision nodes addressing role, risk, expert validation, transparency, and data protection.

To operationalize the proposed principles, we introduce a structured ethical decision pathway designed to guide researchers, supervisors, and research ethics committees in evaluating proposed uses of generative artificial intelligence in sport pedagogy research. The pathway structures ethical deliberation through decision nodes, with each stage addressing distinct dimensions of responsible AI integration (Figure 1).

### 4.1 Stage 1: role assessment

#### 4.1.1 Decision point

Is the proposed AI use peripheral (e.g., grammar checking, reference formatting) or central (e.g., generating instructional feedback, designing practice activities, analyzing movement data)?

If the use is peripheral, the evaluation proceeds to Stage 2. If the use is central, heightened scrutiny is required, and the evaluation proceeds to Stage 2 with recognition that subsequent stages carry greater ethical weight.

### 4.2 Stage 2: risk evaluation

#### 4.2.1 Decision point

Could inaccuracies in AI output affect learner safety, compromise skill acquisition processes, or threaten research validity?

If no substantive risk is identified, evaluation advances to Stage 3. If such risks are present, expert review becomes mandatory.

### 4.3 Stage 3: expert validation

#### 4.3.1 Decision point

Will AI outputs undergo systematic review by qualified domain experts (motor learning specialists, experienced coaches) before implementation?

If such expert validation is in place, the evaluation moves to Stage 4. If expert review is absent or the available expertise is insufficient, the proposed AI use requires modification and may be ethically inappropriate.

### 4.4 Stage 4: transparency

#### 4.4.1 Decision point

Can AI use be transparently disclosed to participants, oversight bodies, and in scholarly outputs, including scope of use and human review procedures?

If transparent disclosure is feasible, the evaluation continues to Stage 5. If transparency cannot be ensured, the proposed AI use may violate informed consent requirements and should be reconsidered.

### 4.5 Stage 5: data protection

#### 4.5.1 Decision point

Does the process adequately protect identifiable learner data (video, biomechanics, performance metrics) including compliance with applicable data protection legislation and explicit informed consent?

If adequate protections are in place, the proposed AI use may be considered ethically defensible, subject to institutional approval. If data protection measures are insufficient, the proposed AI use requires modification or alternative methodological approaches.

Negative responses at any stage indicate ethical concerns requiring resolution before AI implementation. The pathway is sequential because later stages presume earlier conditions are met. Transparency is insufficient if safety risks remain unaddressed; data protection cannot compensate for absent expert validation.

To facilitate practical implementation, each decision node in the pathway should be accompanied by a short governance checklist identifying required documentation, responsible personnel, and the corrective action to be taken when a criterion is not met. For example, central AI uses such as movement-feedback generation, or performance-data interpretation should not proceed without prior expert validation, participant disclosure, and documented data-protection safeguards.

## 5 Discussion

The framework advanced here carries distinct implications for multiple stakeholder groups. For researchers, pedagogical validity introduces a new evaluative criterion: AI-supported processes must be assessed not only for transparency and authorship clarity but also for alignment with motor learning principles. For research ethics committees, the framework suggests that generic AI ethics checklists prove insufficient for embodied learning research and that committee composition or consultation mechanisms may need to include discipline-specific expertise. For institutions developing AI governance policies, the analysis highlights the need to move beyond one-size-fits-all approaches toward models that acknowledge disciplinary epistemologies recognizing the distinctive ethical demands of movement-based learning.

The ethical significance of GenAI should also be differentiated across subfields including motor skill coaching, physical education teaching, sports pedagogy support, and biomechanical analysis, as associated risks are not uniform (2, 21, 22). These risks are grounded in well-established motor learning principles related to attentional focus, adaptive variability, and performer-task-environment interactions (9, 12, 14, 27).

A further limitation of the present Perspective is the absence of systematically collected stakeholder evidence from coaches, researchers, students, and research ethics actors who are currently encountering GenAI in practice, despite the fact that responsible governance in education depends not only on formal principles but also on context-sensitive understanding of how technologies are interpreted, adopted, and contested by users (2, 5, 26). Future research should therefore integrate qualitative interviews, survey-based mapping, Delphi consensus methods, and implementation pilots to determine which ethical risks are most salient in authentic settings, which safeguards are realistically actionable, and how pedagogical validity can be assessed in routine institutional review and professional practice (2, 16, 21).

The proposed framework requires empirical validation across several dimensions. First, comparative experimental studies should assess whether AI-generated feedback produces motor learning outcomes equivalent to expert-designed feedback, examining not only performance endpoints but also learning processes (e.g., attentional focus, movement variability patterns, intrinsic motivation). Second, research should investigate whether the pedagogical validity criteria proposed here (attentional focus appropriateness, autonomy support, context sensitivity, variability support) predict learning effectiveness, establishing construct validity for the framework. Third, implementation studies should examine how research ethics committees and institutional review boards operationalize pedagogical validity assessment in practice, identifying barriers to integration of disciplinary expertise in AI ethics review. Fourth, cross-cultural validation is needed to assess framework applicability in diverse educational contexts with varying regulatory structures and pedagogical traditions. Additionally, emerging applications such as AI-supported biomechanical analysis and wearable sensor integration warrant further investigation, particularly regarding data governance and algorithmic bias in performance evaluation.

At an institutional level, these observations suggest that research ethics committees and governance bodies may need to incorporate disciplinary expertise when evaluating AI-assisted pedagogical research. Reliance on generic AI ethics checklists alone may be insufficient in embodied learning domains where feedback precision, contextual sensitivity, and learner safety are ethically central.

Beyond immediate applications in feedback generation and instructional design, emerging technologies raise additional ethical questions requiring future attention. AI-supported biomechanical analysis systems using computer vision and pose estimation are increasingly deployed in physical education and coaching contexts, raising concerns about algorithmic bias in movement assessment and the validity of automated technique evaluation against expert human judgment (28, 30). Similarly, the integration of wearable sensors with AI analytics for real-time performance monitoring creates novel data-governance challenges, including continuous physiological surveillance, third-party data access, and possible use of performance data in selection or employment decisions (18, 29). These applications extend beyond the instructional-feedback focus of the present framework and underscore that sport pedagogy AI ethics remains an evolving domain requiring continued theoretical development and empirical investigation.

## 6 Limitations

This framework is conceptual and has not yet been empirically validated, and several limitations warrant acknowledgment. First, the framework has not been empirically validated through implementation studies examining how researchers, ethics committees, or institutions operationalize pedagogical validity in practice. The feasibility, reliability, and practical utility of the proposed criteria remain empirical questions. Second, the framework grounded in international AI governance standards and motor learning literature reflects predominantly Western research traditions and regulatory contexts. Cross-cultural validation is needed to assess applicability in educational cultures that differ in pedagogical philosophies, institutional structure, and perceptions of learner autonomy. In addition, because this Perspective does not follow a systematic review protocol, the possibility of interpretive selection bias cannot be fully excluded, even though the analysis was anchored in representative and thematically relevant scholarship. Third, the framework focuses primarily on GenAI applications in feedback generation and instructional design. It may therefore require extension to address emerging applications such as AI-supported biomechanical analysis, automated performance prediction, or integration with wearable-sensor ecosystems. Fourth, ongoing technological evolution may require periodic revision of the framework. For this reason, ethical governance should be treated as version-sensitive and revisable, with periodic reassessment whenever AI capabilities, deployment conditions, or data-processing practices materially change. Fifth, the decision pathway (Figure 1) provides structured guidance but cannot eliminate contextual judgment. Its implementation will require adaptation to specific research settings, institutional policies, and regulatory requirements. Finally, the literature base remains limited to the early stage of AI ethics scholarship in sport pedagogy; as empirical research accumulates, the conceptual foundations of the framework may require refinement.

## 7 Conclusion

Generative artificial intelligence offers substantial opportunities for innovation in sport pedagogy research, yet embodied learning environments introduce ethical complexities that are not adequately addressed by generic AI governance frameworks. By integrating research integrity standards, AI ethics in education, and motor learning theory, this conceptual, theory-guided Perspective advances a discipline-specific ethical framework centered on pedagogical validity, a criterion emphasizing alignment between AI-supported processes and established mechanisms of skill acquisition. The framework positions ethical evaluation of GenAI not merely as a matter of transparency or authorship, but as requiring substantive engagement with disciplinary knowledge about how humans learn movement skills. As generative AI becomes increasingly embedded in educational research, discipline-sensitive ethical reflection will be essential to ensure that technological innovation enhances rather than undermines pedagogical integrity, learner autonomy, and scientific responsibility in embodied learning contexts. The value of the present framework lies not in claiming final resolution, but in providing a structured starting point for empirical testing, institutional adaptation, and discipline-specific refinement. Responsible integration of GenAI in sport pedagogy will depend on linking ethical principles to observable pedagogical consequences, documented human oversight, and continuously updated governance procedures.

## Data Availability

The original contributions presented in the study are included in the article/supplementary material, further inquiries can be directed to the corresponding authors.
